# Validation of a robust method for quantification of three‐dimensional growth of the thoracic aorta using deformable image registration

**DOI:** 10.1002/mp.15496

**Published:** 2022-02-17

**Authors:** Zhangxing Bian, Jiayang Zhong, Jeffrey Dominic, Gary E. Christensen, Charles R. Hatt, Nicholas S. Burris

**Affiliations:** ^1^ Department of Radiology University of Michigan Ann Arbor MI USA; ^2^ Department of Electrical Engineering and Computer Science University of Michigan Ann Arbor MI USA; ^3^ Department of Electrical and Computer Engineering University of Iowa Iowa City Iowa USA; ^4^ Imbio LLC Minneapolis Minnesota USA; ^5^ Department of Biomedical Engineering University of Michigna Ann Arbor MI USA

**Keywords:** deformable registration, thoracic aortic aneurysm, vascular deformation mapping

## Abstract

**Purpose:**

Accurate assessment of thoracic aortic aneurysm (TAA) growth is important for appropriate clinical management. Maximal aortic diameter is the primary metric that is used to assess growth, but it suffers from substantial measurement variability. A recently proposed technique, termed vascular deformation mapping (VDM), is able to quantify three‐dimensional aortic growth using clinical computed tomography angiography (CTA) data using an approach based on deformable image registration (DIR). However, the accuracy and robustness of VDM remains undefined given the lack of ground truth from clinical CTA data, and, furthermore, the performance of VDM relative to standard manual diameter measurements is unknown.

**Methods:**

To evaluate the performance of the VDM pipeline for quantifying aortic growth, we developed a novel and systematic evaluation process to generate 76 unique synthetic CTA growth phantoms (based on 10 unique cases) with variable degrees and locations of aortic wall deformation. Aortic deformation was quantified using two metrics: area ratio (AR), defined as the ratio of surface area in triangular mesh elements and the magnitude of deformation in the normal direction (DiN) relative to the aortic surface. Using these phantoms, we further investigated the effects on VDM's measurement accuracy resulting from factors that influence the quality of clinical CTA data such as respiratory translations, slice thickness, and image noise. Lastly, we compare the measurement error of VDM TAA growth assessments against two expert raters performing standard diameter measurements of synthetic phantom images.

**Results:**

Across our population of 76 synthetic growth phantoms, the median absolute error was 0.063 (IQR: 0.073–0.054) for AR and 0.181 mm (interquartile range [IQR]: 0.214–0.143 mm) for DiN. Median relative error was 1.4% for AR and 3.3% for DiN at the highest tested noise level (contrast‐to‐noise ratio [CNR] = 2.66). Error in VDM output increased with slice thickness, with the highest median relative error of 1.5% for AR and 4.1% for DiN at a slice thickness of 2.0 mm. Respiratory motion of the aorta resulted in maximal absolute error of 3% AR and 0.6 mm in DiN, but bulk translations in aortic position had a very small effect on measured AR and DiN values (relative errors <1%). VDM‐derived measurements of magnitude and location of maximal diameter change demonstrated significantly high accuracy and lower variability compared to two expert manual raters (p<0.03 across all comparisons).

**Conclusions:**

VDM yields an accurate, three‐dimensional assessment of aortic growth in TAA patients and is robust to factors such as image noise, respiration‐induced translations, and differences in patient position. Further, VDM significantly outperformed two expert manual raters in assessing the magnitude and location of aortic growth despite optimized experimental measurement conditions. These results support validation of the VDM technique for accurate quantification of aortic growth in patients and highlight several important advantages over diameter measurements.

## 1 INTRODUCTION

The thoracic aorta is the largest artery in the body, carrying blood from the heart to the rest of the systemic circulation. A variety of degenerative and inflammatory processes cause the degradation of the structural integrity of the normally elastic aortic wall, resulting in thoracic aortic aneurysm (TAA). Aneurysms of the thoracic aorta are often asymptomatic and indolent, either remaining stable or growing slowly over a period of years or decades; however, a small fraction of patients experience acute complications^1^ such as rapid growth, aortic dissection, or aortic rupture, all of which necessitate urgent surgical repair and are potentially fatal. Current clinical guidelines recommend routine imaging surveillance of TAA, and surveillance regimens typically consist of annual or biannual computed tomography angiography (CTA) examinations to assess for interval growth for other aortic complications. Maximal aortic diameter is the primary metric that is used to assess growth and determine candidacy for surgical repair, with measurements typically performed either manually or in a semiautomated fashion using analysis software that allows for multiplaner or centerline‐based measurements in planes orthogonal to the aortic axis.

Despite optimal measurement technique and operator experience, current diameter measurement techniques are associated with substantial measurement variability—on the order of ±2–5 mm—often limiting confident assessment of aortic growth at typical aortic growth rates (<1 mm per year).^2^, ^3^ There are many potential sources of error/variability with diameter measurements. Common issues involve differences in measurement location along the length of the aorta, differences in angulation of the two‐dimensional (2D) measurement planes, and differences in radial position of the diameter calipers (especially when the aortic cross section is noncircular/elliptical). Without improved methods to measure aortic growth, confident determination of disease progression, accurate assessment of patient risk, and fully informed treatment decisions will not be possible.

To address this problem, our group has recently proposed a method, termed vascular deformation mapping (VDM),^4^ to quantify aortic growth in a more accurate and comprehensive fashion. This approach employs deformable image registration (DIR) to quantify three‐dimensional (3D) changes in the aortic wall morphology using high‐resolution volumetric CTA data. Preliminary reports in a clinical population of patients with TAA have shown that the VDM technique may be useful for more complete depiction of the extent of aortic growth to inform surgical planning and for the assessment of growth during imaging surveillance.^4^, ^5^ However, the VDM approach and key algorithms have not yet been validated in a manner that supports the improved accuracy of VDM‐derived measurements compared to standard diameter assessments. B‐spline based techniques for DIR are well‐established and can achieve submillimeter registration accuracy using clinical CT data.^6^ However, a variety of factors related to physiologic motion and image reconstruction may influence the accuracy of registration results between serial aortic CTA examinations, and thus a comprehensive evaluation of the influence of these factors is warranted. Furthermore, determining the performance of aortic measurement techniques (diameter or VDM) using only clinical CT data is severely limited by the inability to determine ground truth aortic growth. Alternatively, phantom experiments provide a unique opportunity to precisely define the degrees and locations of aortic growth.

The objectives of this study were threefold: (1) to determine the accuracy of our VDM pipeline for measuring deformation of the aortic wall in TAA using a representative sample of synthetically generated CTA phantom pairs; (2) examine the influence of a variety of variables that influence clinical CTA data (e.g., respiratory motion, slice thickness, and image noise) on the accuracy of the VDM‐derived deformation assessment; and (3) compare the accuracy of growth measurements between VDM and experienced manual raters using synthetic phantoms to better quantify the potential benefit on clinical growth assessments.

## 2 METHODS

This section describes the VDM registration pipeline and the procedure to create the synthetically deformed images used in this study. The validation procedure for assessing the accuracy of VDM‐based maximal diameter change measurements compared with ground truth is also described.

### 2.1 VDM registration

**FIGURE 1:**
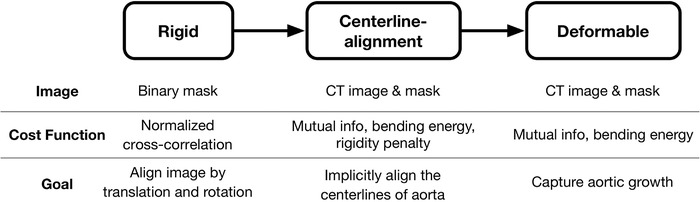
The registration pipeline

Aortic segmentation was performed manually using segmentation software (Mimics, version 22.0; Materialise) as previously described.^4^ All images were precropped from just above the aortic arch through the upper abdomen (i.e., celiac artery). The average volume size is 230 ×230 ×440 with a voxel spacing of 0.64 ×0.64 ×0.75 mm${}^{3}$. All negative HU values are clamped to zero to avoid the influence of lung tissue. Given two serial CT images with corresponding aortic segmentation masks, we use the VDM pipeline, as shown in Figure 1, to measure the growth of the aortic wall.^4^ The registration consists of three main steps: rigid registration, aortic centerline alignment, and deformable registration.

The rigid registration uses segmentations of the aorta to rigidly align the images based on the normalized cross‐correlation metric. Given a transformation parameterized by μ, the normalized cross‐correlation (NCC) is defined as

(1) $$\begin{array}{ccc} & & \mathrm{NCC}\left(\mu \right)=\\ & & \frac{\sum_{x_{i}\in \Omega_{F}}\left(I_{F}\left(x_{i}\right)-\bar{I_{F}}\right)\left(I_{M}\left(T_{\mu }\left(x_{i}\right)\right)-\bar{I_{M}}\right)}{\sqrt{\sum_{x_{i}\in \Omega_{F}}{\left(I_{F}\left(x_{i}\right)-\bar{I_{F}}\right)}^{2}\sum_{x_{i}\in \Omega_{F}}{\left(I_{M}\left(T_{\mu }\left(x_{i}\right)\right)-\bar{I_{M}}\right)}^{2}}}, \end{array}$$

where $\bar{I_{F}}=\frac{1}{|\Omega_{F}|}\sum_{x_{i}\in \Omega_{F}}I_{F}\left(x_{i}\right)$ and $\bar{I_{M}}=\frac{1}{|\Omega_{F}|}\sum_{x_{i}\in \Omega_{F}}I_{M}\left(T_{\mu }\left(x_{i}\right)\right)$ indicate the average value of fixed image and transformed moving image.

The centerline alignment and deformable registration steps both use a multiimage, multicost function strategy, with each pair of images focusing on a different cost. Centerline alignment is a DIR step that is highly regularized by bending energy^7^ and aortic rigidity penalties,^8^ which implicitly registers the aortic centerlines by allowing nonrigid movement of the tissues adjacent to the aorta but a rigid movement of the aorta itself.

Bending energy is defined as

(2) $$P_{\mathrm{BE}}\left(\mu \right)=\frac{1}{N}\sum_{{\tilde{x}}_{i}\in N}{\left\|\frac{\partial^{2}T}{\partial x\partial x^{T}}\left({\tilde{x}}_{i}\right)\right\|}_{F}^{2},$$

where *N* is the size of neighbor set N. A bending energy penalty is used in VDM to regularize DIR by penalizing the high‐frequency changes in the deformation field and also help avoid folding artifacts.

A rigidity penalty is used to enforce local rigidity of the deformation field by penalizing local compression/expansion and deviations from linearity (LN), orthonormality (OC), and properness (PC) of the deformation field Jacobian^8^:

(3) $$\begin{array}{ccc} P_{\mathrm{rigid}}\left(\mu \right) & = & \frac{1}{\Sigma_{x}c\left(x+u\left(x\right)\right)}\sum_{x}c\left(x+u\left(x\right)\right)\left\{\mathrm{LN}{\left(x\right)}^{2}\right.\\ & & \left.+\;\mathrm{OC}{\left(x\right)}^{2}+\mathrm{PC}{\left(x\right)}^{2}\right\}, \end{array}$$

where the rigidity coefficient is set to 0 for a pixel that corresponds to nonrigid tissue and to 1 for rigid tissue. In our case, the aortic mask is dilated by five voxels to serve as rigidity coefficient map.

Using both bending energy and rigidity penalty allows the final DIR step to (1) focus primarily on aortic growth via measurement of wall deformation and (2) reduce the need for a large capture range. The centerline alignment utilizes one similarity metric (mutual information, MI), and two regularization penalties (bending energy with weight of 10 and rigidity with weight of 20). MI^9^ is a widely used metric that had originally been developed for multimodality registration.^10^ In our initial experiments,^5^ we found MI to produce the most accurate results in comparison to other metrics such as normalized cross‐correlation and a sum of squared differences, presumably because MI implicitly focuses on the alignment of boundaries as well as the fact that the intensity of the intraluminal iodine contrast agent can vary between CTA scans.

The final DIR step performs B‐spline‐based registration on a finer grid (0.48×0.48×0.625 mm${}^{3}$) and with MI as similarity measurement and uses a larger bending energy term (with a weight of 100) than the centerline alignment step to align the aortic wall between the baseline and follow‐up images. The displacement field used for further steps is generated from the final deformable registration step. Our workflow is implemented in Elastix.^11^

### 2.2 Generation of synthetically deformed CTA images

#### 2.2.1 Step 1: Manually deformed aortic mesh modeling

A 3D surface was built using the Marching Cubes algorithm^12^ applied to an aortic segmentation of the fixed CT image. We used an open‐source 3D modeling software (Blender, http://www.blender.org) to perform deformation of the aortic surface and create synthetic aortic growth phantoms. Each mesh was defined as a set of vertices $V=v_{1},v_{2},\text{…},v_{N}$, and each face, $f_{\left\{v_{i},v_{j},v_{k}\right\}}$, was constructed by grouping three neighboring vertices. Each vertex $v_{i}$ has a position $\left(x_{i},y_{i},z_{i}\right)$ in the 3D space. We denote the vertices in deformed surface as $\tilde{V}$; vertex‐wise correspondence is maintained during the manual‐deform process, that is, $v_{i}↔\tilde{v_{i}},v_{i}\in V,\tilde{v_{i}}\in \tilde{V}$.

**FIGURE 2:**
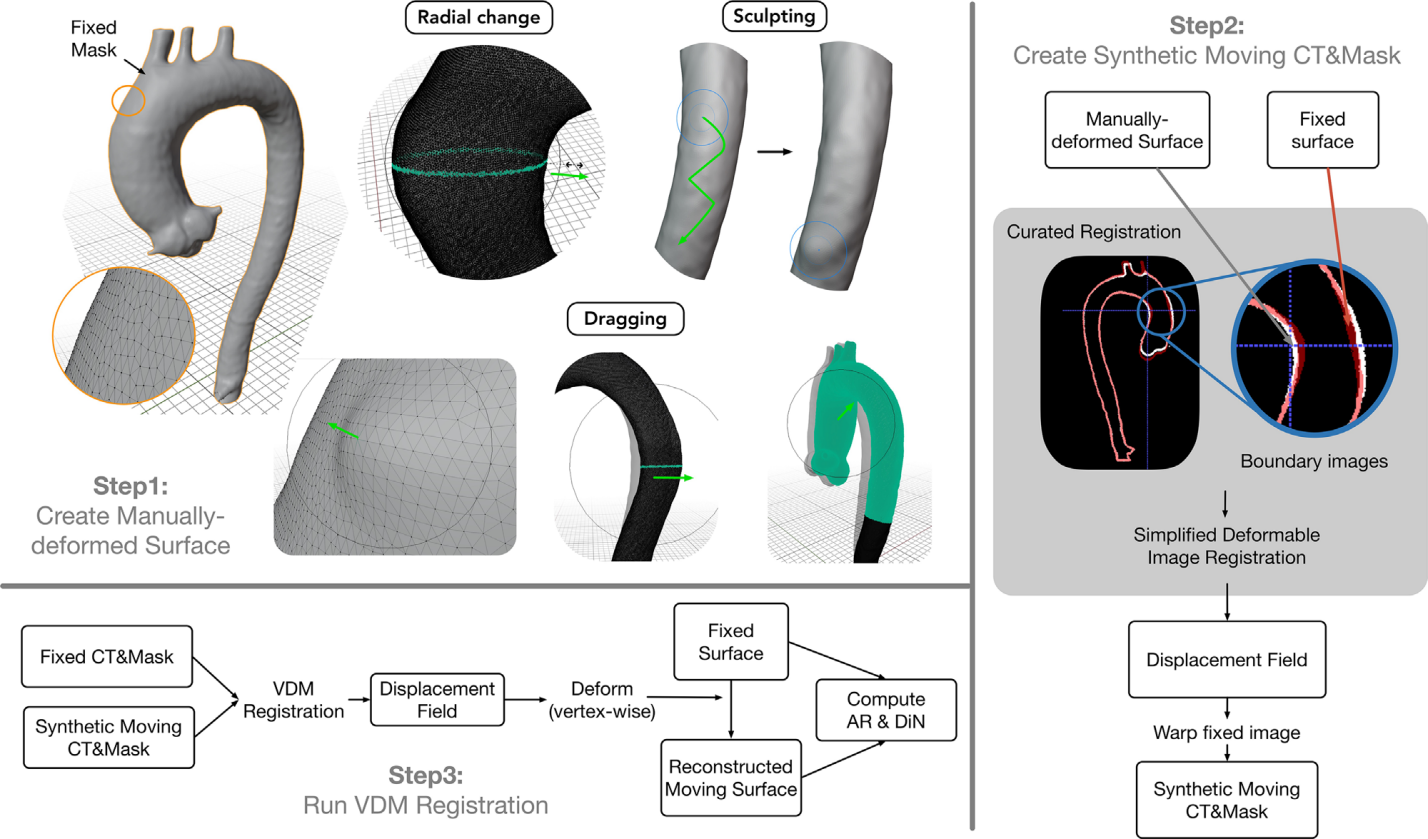
Pipeline for creating synthetic images and validation process. Step 1: three techniques are used to create the deformations on 3D meshes: radial change, sculpting, and dragging. Step 2: a single‐step curated DIR registration is used to align the fixed and manually deformed surfaces. Subsequently, the resulting transformation is used to warp the fixed CT and mask to obtain the synthetic moving images. The displacement field is used to deform the fixed surface to create the synthetic moving surface. Step 3: VDM is used to register the synthetic moving images with fixed images and compute the metrics: AR and DiN

All synthetic growth phantoms were derived from high‐quality, electrocardiogram‐gated CTA scans of the thoracic aorta acquired on a single CT scanner (Discovery CT750 HD, GE Healthcare) with the following parameters: 100 kVP, tube current 340–480 mA, pitch 1.375:1, Noise index 19.84, average CTDIvol of 3.78, large body, using 95‐mL iopamidol 370 mg I/mL (Isovue 370, Bracco Diagnostics, Inc., Princeton, NJ, USA) injected at 4 mL/s, followed by a 100‐mL saline chaser at 4 mL/s with axial reconstructions at 0.625 mm section thickness and 0.625 mm intervals at 75% of the cardiac cycle. Synthetic deformations were manually created with variable locations along the aorta and magnitudes under the guidance of an experienced cardiothoracic radiologist (N.S.B.) and were designed to simulate clinically observed aortic shapes and growth patterns. Three primary modes of growth were utilized to create growth phantoms (as depicted in Figure 2, Step 1):

- Outward radial deformation along the circumference of an aortic cross section, which mimics typical fusiform growth.
- Sculpting, which mimics an irregular region of eccentric/saccular bulging often seen in association with atherosclerotic plaque.
- Dragging a group of vertices to simulate bending and/or stretching. Specifically, we used this operation to simulate respiratory‐related aortic translations.

**FIGURE 3:**
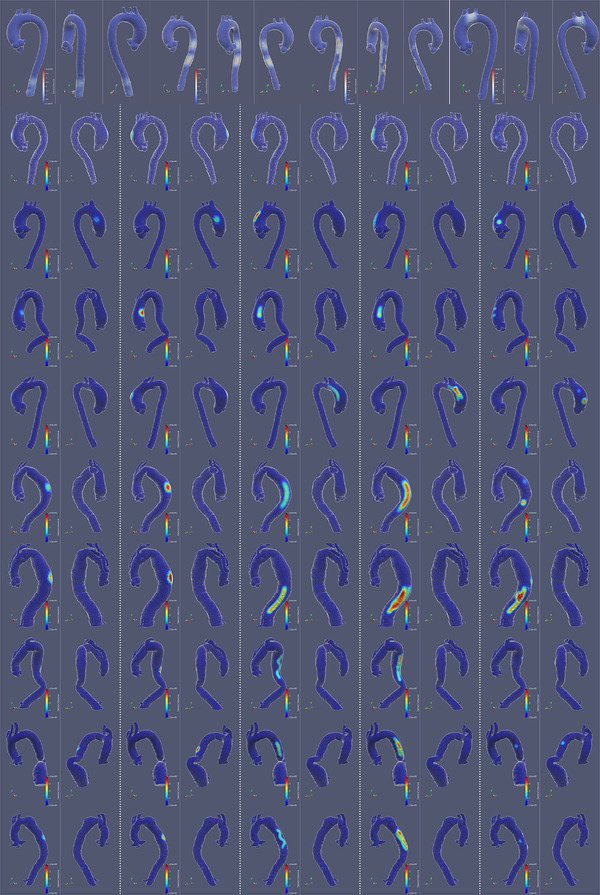
Gallery of synthetic deformation. The first row shows some examples from 31 deformations based on one case. For the other nine rows, each row shows five deformations (columns) based on a case, each with two views. The deformed surface is shown in white wireframe overlayed on fixed surface. The heatmap of deformation (in normal direction) is plotted on the fixed surface

An image gallery depicting synthetic deformations is shown in Figure 3.

#### 2.2.2 Step 2: Synthetic moving image and mesh creation

Following creation of the original and deformed meshes (defined by V and $\tilde{V}$), synthetically deformed CT images and aortic segmentation masks are generated. This is done by using V and $\tilde{V}$ to create “boundary” images (B and $\tilde{B}$) , which are then registered to create a deformation field, and we consider this deformation field as the ground truth for all further experiments.

**FIGURE 4:**
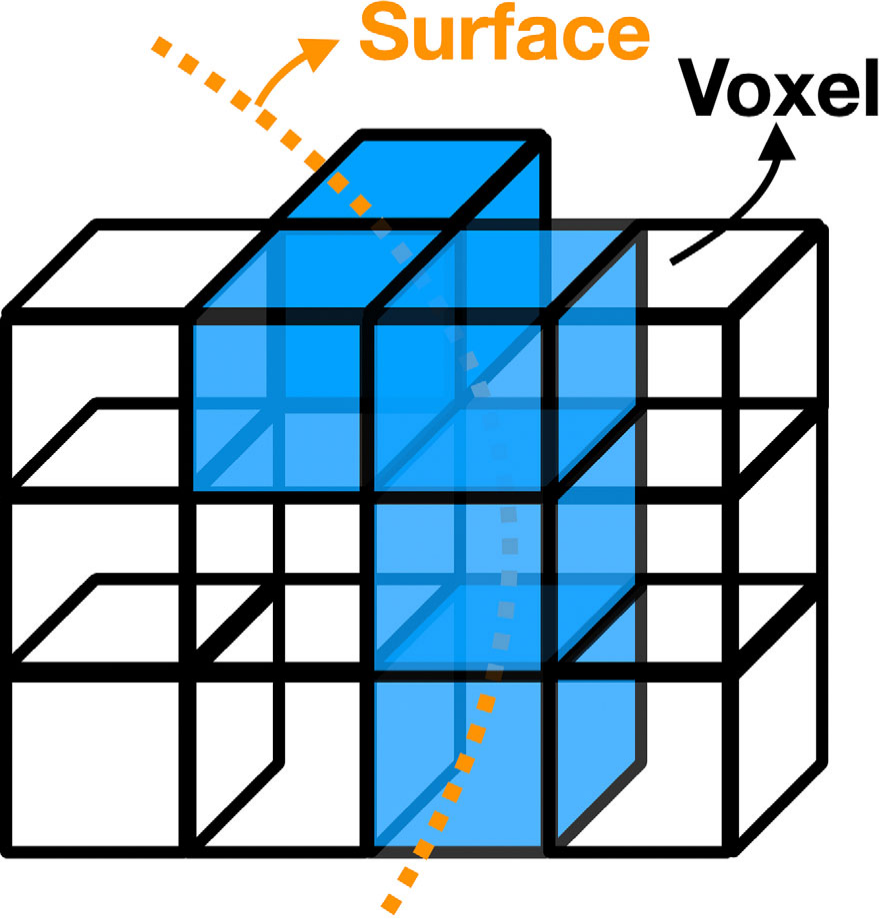
Illustration of how a surface mesh is converted to a boundary image. Voxels that are occupied by mesh vertices (shaded blue) are set to one, all others are zero

Specifically, in the “boundary image,” voxels that occupy any vertex are set to one and are otherwise zero, as shown in Figure 4. We applied Gaussian blurring with sigma = 5 on the binary image to soften the boundary and facilitate the following registration step. Then we register these two boundary images with B as the moving image and $\tilde{B}$ as the fixed image, using a simplified (single‐step) B‐spline‐based deformable registration. The resulting deformation fields are used to create a deformed CTA aorta mask $\hat{M}$ by the *transformix* tool in *Elastix*, and a new set of vertices defining a third mesh $\hat{V}$. Note that $\hat{V}$ rather than $\tilde{V}$ represents an aortic surface that is perfectly concordant with the anatomy shown in the synthetic moving $\hat{I}$ and the simulated deformation field. A schematic depiction of this workflow is shown in Figure 2, Step 2.

#### 2.2.3 Step 3: Registration‐based VDM analysis

**FIGURE 5:**
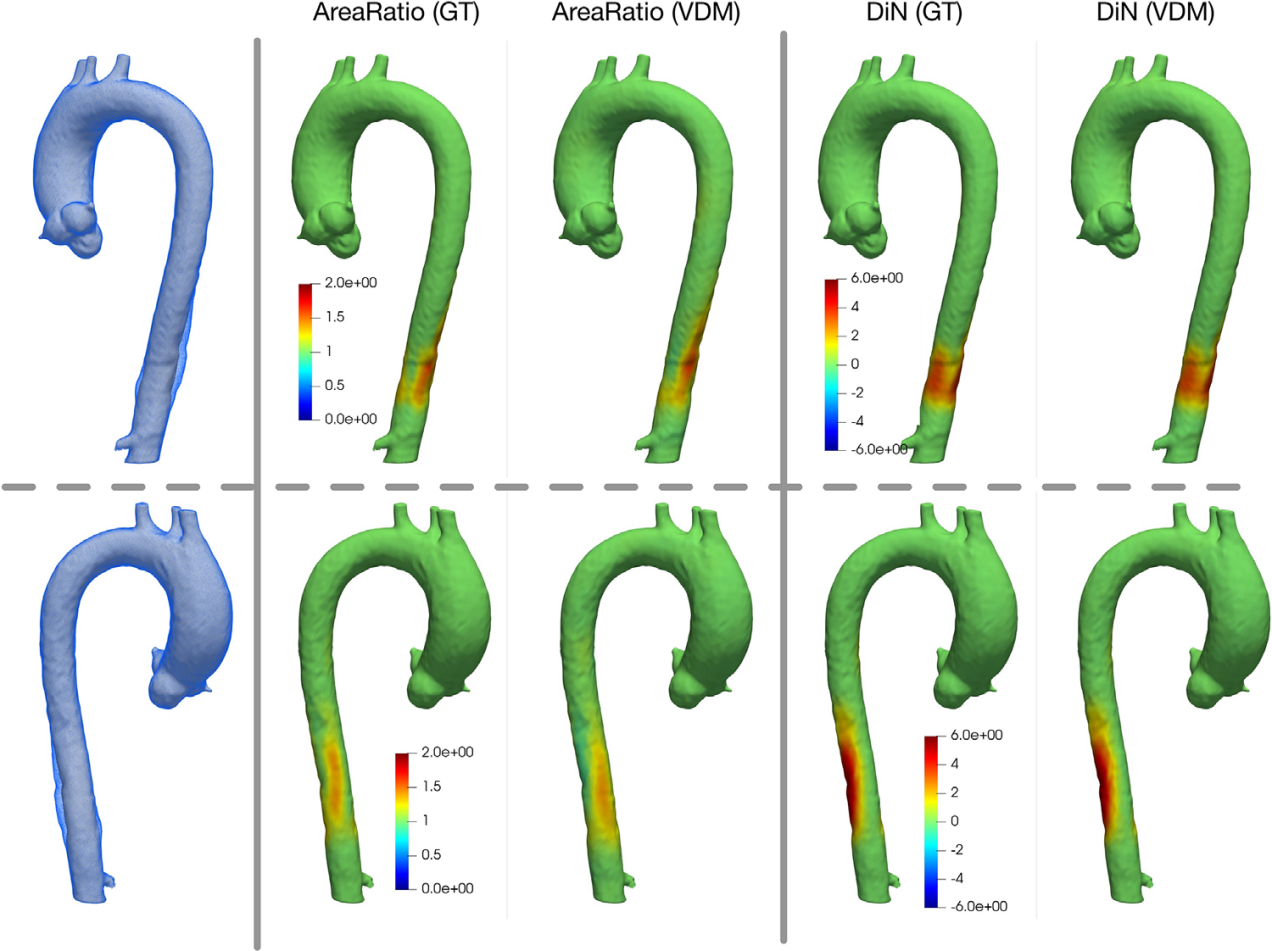
Examples of the ground‐truth and VDM‐based AR and DiN metrics for growth quantification shown for a representative synthetic phantom case. The white solid surface is the fixed surface, and the blue semitransparent surface is the synthetic moving surface

After generation of the synthetic moving CTA image and mask from Step 2, we register it with the fixed image through the full VDM pipeline (Figure 1) and deform the fixed surface using the deformation field (resulting from the VDM). Then we compute the ratio of change in surface area at each triangular mesh element, termed area ratio (AR) and the magnitude of deformation in the normal direction (DiN) relative to the aortic surface. To visualize the results, we interpolate the quantitative growth metrics onto the vertices of fixed surface; a representative example case from our synthetic phantom cohort demonstrating our quantitative aortic growth metrics is shown in Figure 5. The computation of AR and DiN are explained in Section.2.3.1.

### 2.3 Validation

#### 2.3.1 Quantitative growth metrics

**FIGURE 6:**
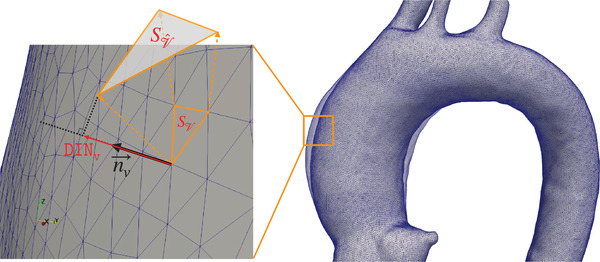
An illustration for computing DIN (i.e., Equation 5) and AreaRatio (i.e., Equation 4)

We define two mesh‐based metrics for measuring aortic growth: AR and deformation in the normal direction to the aortic mesh surface (DiN), as shown in Figure 6. AR is defined as the ratio of the area of a face in one mesh (e.g., moving surface) to that of the corresponding face in another mesh (e.g., moving surface).

(4) $$AR_{f}=\frac{S_{\hat{V}}}{S_{V}}=\frac{S(f_{\left\{\hat{v_{i}},\hat{v_{j}},\hat{v_{k}}\right\}})}{S(f_{\left\{v_{i},v_{j},v_{k}\right\}})},$$

where the S(·) computes the area for a given face.

The DiN metric, which is computed at each mesh vertex and defined in Equation (5), is computed by projecting registration‐derived displacement vectors between two corresponding vertices (one on the fixed surface and another on moving surface) onto the corresponding normal vector on the fixed surface mesh. This metric reflects the magnitude of deformation (in millimeters) perpendicular to the aortic surface at each vertex:

(5) $$DiN_{v_{i}}={\vec{n}}_{v_{i}}\cdot \left(v_{i}-\hat{v_{i}}\right).$$

**FIGURE 7:**
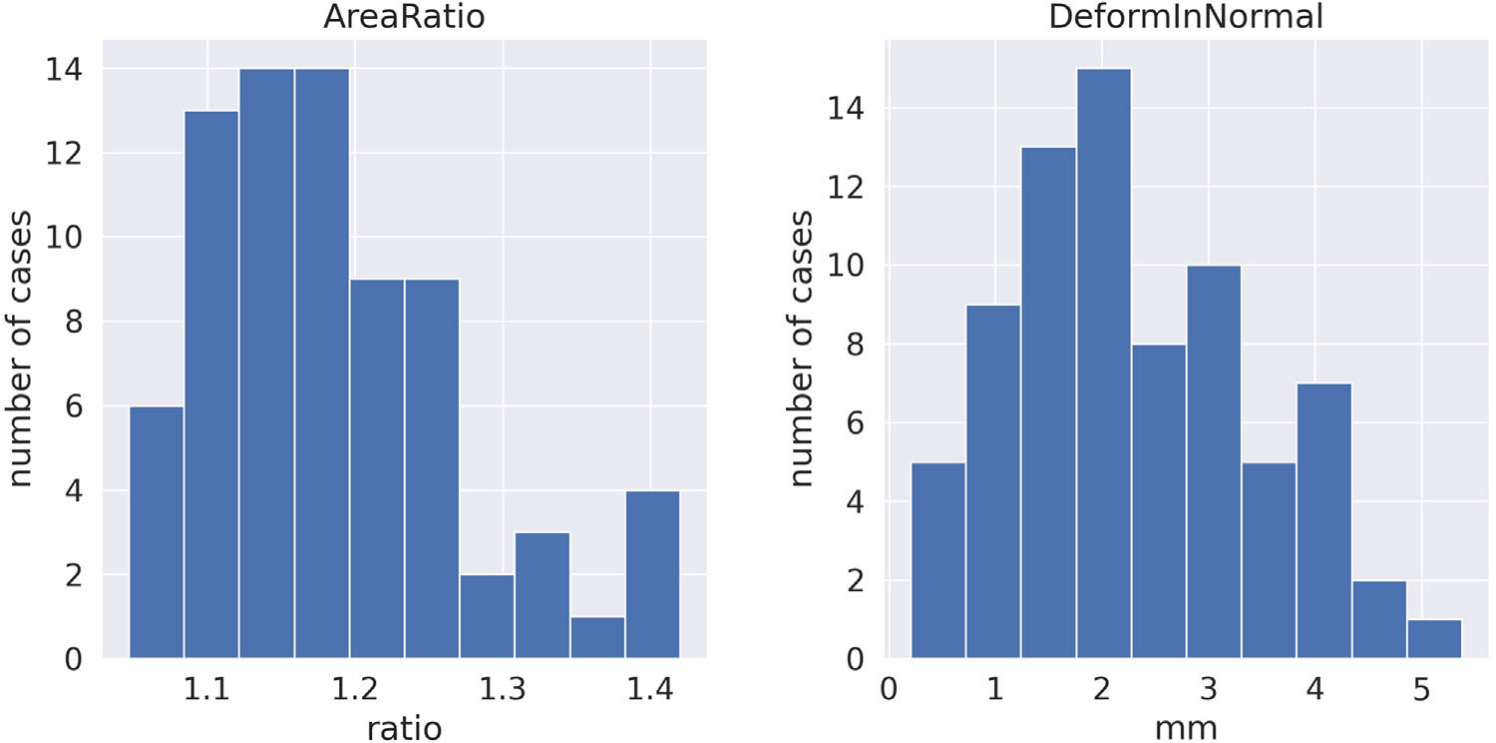
Histograms depicting the 99th percentile value of synthetic deformations for AR and DiN metrics. The 99th percentile values are computed without considering the faces and vertices with deformation magnitudes smaller than a threshold of 0.01 mm. The (25th, 50th, 75th) percentile of for AR and DiN across all cases are (1.12, 1.17, 1.23 mm) and (1.49, 2.03, 3.06), respectively

Histograms depicting the distribution of DiN and AR values in all synthetic deformations across our 76 phantom population are shown in Figure 7.

#### 2.3.2 Validation of quantitative measurement robustness

**FIGURE 8:**
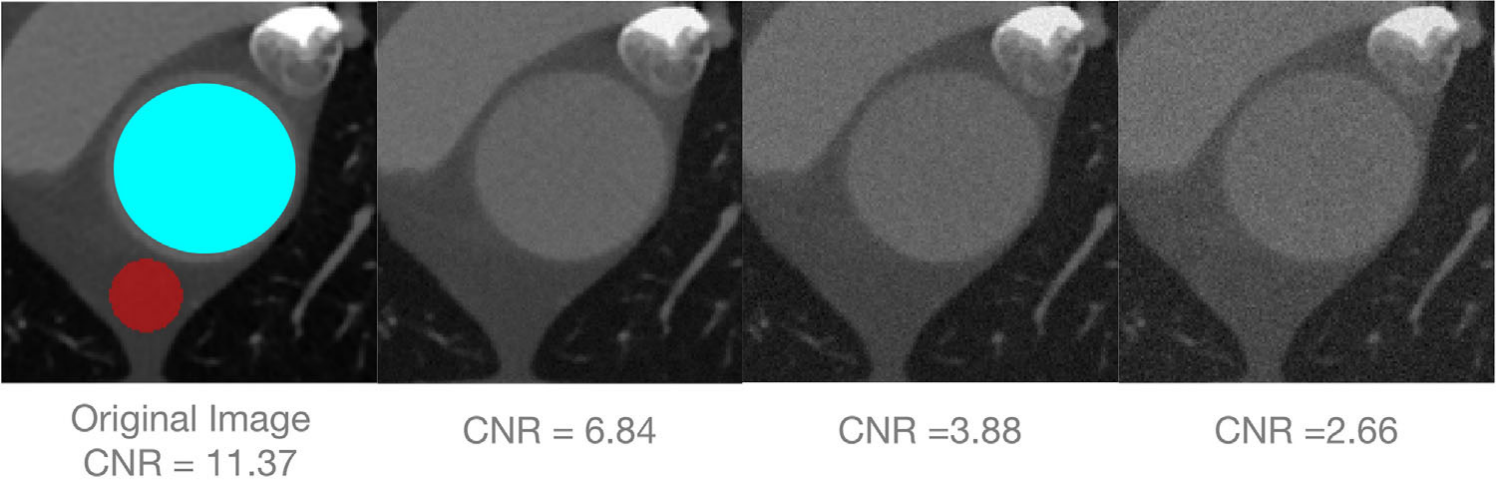
CNR computation. Manually generated ROIs were placed within the mediastinal fat (red) to compute background HU statistics, while the aortic segmentation mask was eroded by three pixels to create an ROI (cyan) used for computation of aortic HU statistics

The robustness of VDM growth quantification using AR and DiN metrics was assessed for a variety of factors that may affect registration accuracy including slice thickness, image noise, and bulk patient motion. The effect of image noise was tested by adding add various magnitudes of Gaussian noise (50 HU, 100 HU, and 150 HU) to the CT images before performing registration, corresponding to contrast‐to‐noise ratios (CNRs) of 6.84, 3.88, 2.66, respectively. CNR was computed using the following equation:

(6) $$\mathrm{CNR}=\frac{\mathrm{Contrast}}{\mathrm{Noise}}=\frac{\left|\mu_{\text{aorta}}-\mu_{bg}\right|}{\sqrt{\sigma_{\text{aorta}}^{2}+\sigma_{bg}^{2}}},$$

where $\mu_{\text{aorta}},\mu_{bg},\sigma_{\text{aorta}}\sigma_{bg}$ are the means and standard deviations of the HU values in regions of interest in the aorta and adjacent mediastinal fat, respectively. More details regarding the CNR calculation are shown in Figure 8.

**FIGURE 9:**
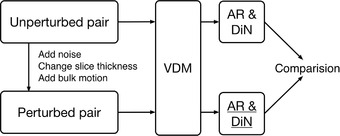
Workflow of the robustness test

The effect of CT slice thickness on AR and DiN was also tested at three different slice thicknesses representative of a range typically used for clinical CTA: 1.0, 1.5, and 2.0 (mm). We tested the effect of patient bulk motion by randomly rotating (according to a uniform distribution {+5,−5} degrees) and translating the image by {20,40,60} (mm) along three axes. For each level of these factors (i.e., noise, slice thickness, and bulk motion), a pair of perturbed fixed and moving synthetic images were created. The full VDM analysis pipeline was performed, and the resulting AR and DiN values were compared to unperturbed results by calculation of absolute and relative errors. A schematic depicting this workflow is shown in Figure 9.

Finally, while clinical CTA is most often acquired during inspiration, we tested the effect of respiratory motion of the aorta and how serial CTA scans acquired at different phases of respiration would affect the accuracy of VDM growth measurements. To do this, an additional six synthetic moving images were created that had a combination of localized deformation of the aortic wall in addition to differences in the respiratory position of the aorta based on published values.^13^ Specifically, we selected six cases with varying degrees and locations (e.g., ascending and descending) of growth and used Blender's dragging tool (Figure 2) to translate the ascending aortic, arch, and proximal descending aorta in a physiologically realistic manner.

#### 2.3.3 Maximal diameter measurement: Expert manual measurements versus VDM

In this section, we focus on the typical clinical task, measuring the maximal aortic diameter change (i.e., growth) and describe the procedure used to compare VDM‐based growth measurements against manual measurements.

Two independent, expert raters (advanced image analysis technologists) with 5 years (Rater 1) and 15 years (Rater 2) of experience with aortic measurements, identified the location where the maximum diameter change happens and measured the change according to a standard workflow: each rater viewed the synthetically deformed and original CTA images side‐by‐side and attempted to locate the position where the maximum deformation occurred.

Given that the deformed moving image was synthetically created from the original image, the anatomy was intrinsically registered except at the local region of deformation, which made this task easier than in a real‐life clinical scenario where changes in patient positioning and the positioning of adjacent organs makes a visual comparison of side‐by‐side images more difficult. Thus, the rater's performance on the synthetic cases was considered the best case scenario for what can be achieved with routine manual measurements.

The ground truth maximal diameter change was measured by first extracting the aortic centerline of the fixed image then sampling the centerline at points every 0.5 mm. The maximum diameter of each cross section (orthogonal to the centerline) was then computed by the open‐source Vascular Modeling Toolkit (VMTK, http://www.vmtk.org).^14^ We denote the results as two one‐dimensional (1D) arrays $d_{V_{\text{fixed}}}$ and $d_{V_{\text{smoving}}}$, with each having the length equal to the number of point samples on the centerline. Then we take $\max(|d_{V_{\text{fixed}}}-d_{V_{\text{smoving}}}|)$ as the ground truth maximal diameter change and record the location of the maximal diameter change along the centerline.

In the VDM‐based diameter measurement, we obtained the reconstructed moving surface by deforming the fixed surface using the displacement field resulting from the registration step. Similarly, we take the same sampled centerline and measure the maximum diameter at each centerline point for both reconstructed moving surface and fixed surface and record the magnitude and location of the largest change in diameter.

##### Statistical analysis

We performed a priori sample size estimates for our manual rater experiments using an *F*‐test of variances and assuming a conservative standard deviation of measurement error of ±0.3 mm for VDM (based on preliminary experiments) and standard deviation of manual aortic diameter measurements of ±1 mm from prior literature.^15^ This calculation showed a 99% power to detect a difference between groups with a sample size of *n* = 30 synthetic phantoms. Levene's test was used to examine differences in variance of errors, and the Wilcoxon test was used to examine group differences in absolute errors. A *p*‐value of <0.05 was considered significant for all statistical tests. Statistical analyses were performed using Stata 14.0 (StataCorp LP, College Station, TX, USA).

## 3 RESULTS

### 3.1 Comparison between VDM and ground truth growth metrics

**FIGURE 10:**
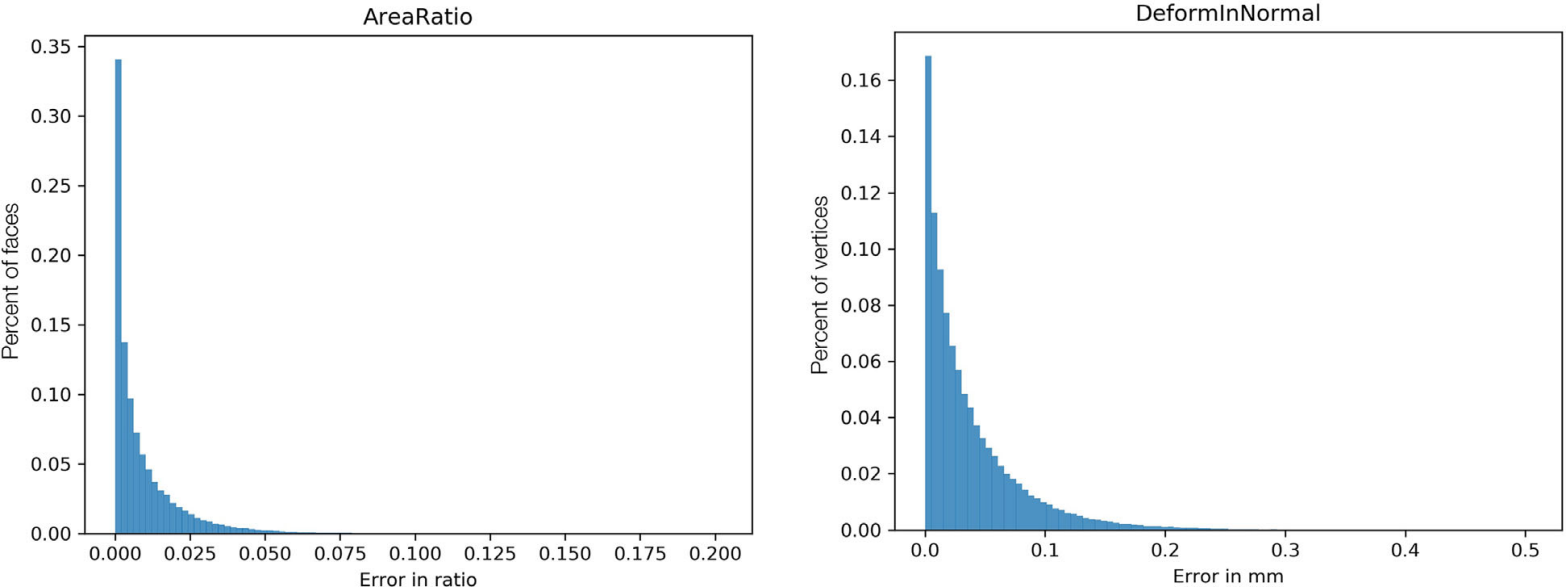
The histogram showing the absolute error across all mesh elements for all cases. Each data point in histogram represents an error for a vertex (DiN) or a face (AR)

Across our population of 76 synthetic growth phantoms, the median absolute error was 0.063 (interquartile range [IQR]: 0.073–0.054) for AR and 0.181 mm (IQR: 0.214–0.143 mm) for DiN. Absolute error for AR and DiN showed a moderate positive correlation with the degree of maximal aortic deformation (AR: *R* = 0.29; DiN: *R* = 0.37). There was a small but statistically significant difference in the median absolute error between cases of ascending versus descending TAA for AR (median ascending 0.060, IQR: 0.044–0.065 vs. median descending 0.071, IQR: 0.060–0.076; p<0.001); however, there was not a statistically significant difference for DiN (median ascending 0.171, IQR: 0.131–0.211 vs. median descending 0.185, IQR: 0.159–0.208; *p* = 0.342). Figure 10 shows a summary of mesh element‐wise error across all cases, with summary statistics of errors for each of the 76 cases displayed in Table 1.

**TABLE 1:** Detailed error statistics for each case within the deformed region (defined deformation magnitude larger than 1e‐3 mm)

			Deformation in normal direction (DiN) error					Area ratio (AR) Error				
Case ID	Maximum deformation location	Maximum deformation in normal direction (GT)	99th Perc.	95th Perc.	Median	Mean	Std.	99th Perc.	95th Perc.	Median	Mean	Std.
1	Descending	1.861	0.168	0.159	0.061	0.125	0.024	0.073	0.068	0.029	0.033	0.012
2	Descending	1.407	0.130	0.116	0.048	0.096	0.016	0.047	0.042	0.017	0.021	0.007
3	Descending	1.996	0.279	0.262	0.123	0.118	0.067	0.128	0.123	0.058	0.063	0.020
4	Descending	2.377	0.179	0.170	0.082	0.106	0.035	0.047	0.042	0.018	0.022	0.007
5	Descending	2.062	0.237	0.231	0.111	0.143	0.054	0.089	0.084	0.037	0.035	0.016
6	Descending	4.043	0.323	0.321	0.143	0.205	0.044	0.133	0.128	0.064	0.067	0.020
7	Descending	5.379	0.473	0.457	0.226	0.246	0.096	0.132	0.127	0.060	0.061	0.022
8	Descending	2.09	0.298	0.287	0.131	0.153	0.072	0.145	0.139	0.069	0.072	0.022
9	Descending	2.875	0.605	0.595	0.297	0.350	0.122	0.059	0.053	0.023	0.020	0.011
10	Arch	1.443	0.127	0.116	0.056	0.058	0.034	0.045	0.039	0.016	0.019	0.006
11	Arch	0.972	0.116	0.115	0.049	0.065	0.029	0.046	0.041	0.018	0.016	0.008
12	Arch	0.952	0.222	0.219	0.098	0.118	0.063	0.048	0.043	0.020	0.020	0.008
13	Ascending	1.655	0.115	0.109	0.049	0.113	0.021	0.055	0.049	0.021	0.025	0.008
14	Descending	0.77	0.141	0.135	0.066	0.130	0.045	0.045	0.040	0.015	0.015	0.008
15	Ascending	0.391	0.182	0.164	0.075	0.059	0.025	0.086	0.081	0.036	0.036	0.015
16	Ascending	0.203	0.168	0.158	0.079	0.072	0.027	0.073	0.067	0.032	0.028	0.013
17	Descending	1.224	0.164	0.161	0.080	0.088	0.041	0.102	0.097	0.048	0.052	0.015
18	Ascending	0.208	0.177	0.167	0.071	0.134	0.038	0.084	0.079	0.038	0.035	0.015
19	Descending	1.325	0.145	0.131	0.056	0.033	0.043	0.046	0.041	0.020	0.016	0.008
20	Descending	1.981	0.254	0.241	0.119	0.181	0.052	0.116	0.111	0.055	0.052	0.020
21	Ascending	0.734	0.132	0.130	0.056	0.066	0.045	0.047	0.041	0.016	0.013	0.009
22	Descending	0.406	0.328	0.319	0.155	0.215	0.058	0.048	0.042	0.019	0.019	0.008
23	Ascending	0.68	0.135	0.117	0.046	0.102	0.022	0.041	0.036	0.017	0.013	0.008
24	Arch	1.61	0.279	0.271	0.123	0.131	0.061	0.044	0.038	0.019	0.015	0.008
25	Ascending	2.053	0.174	0.583	0.283	0.285	0.125	0.060	0.054	0.028	0.020	0.012
26	Ascending	2.532	0.569	0.553	0.266	0.270	0.111	0.057	0.051	0.025	0.027	0.008
27	Ascending	3.104	0.398	0.394	0.183	0.199	0.078	0.057	0.052	0.026	0.026	0.009
28	Ascending	1.935	0.171	0.168	0.072	0.105	0.027	0.079	0.074	0.034	0.034	0.013
29	Descending	4.042	0.203	0.192	0.091	0.154	0.053	0.065	0.060	0.027	0.024	0.012
30	Ascending	4.14	0.296	0.284	0.127	0.149	0.056	0.085	0.080	0.038	0.042	0.013
31	Ascending	2.806	0.111	0.092	0.038	0.023	0.037	0.052	0.047	0.020	0.021	0.009
32	Ascending	1.487	0.209	0.201	0.081	0.127	0.032	0.073	0.067	0.033	0.035	0.011
33	Ascending	3.107	0.304	0.286	0.126	0.140	0.063	0.084	0.078	0.037	0.037	0.014
34	Ascending	0.99	0.309	0.292	0.138	0.113	0.060	0.064	0.059	0.025	0.021	0.012
35	Ascending	2.304	0.240	0.232	0.096	0.099	0.041	0.071	0.065	0.032	0.036	0.010
36	Ascending	1.051	0.283	0.273	0.135	0.168	0.062	0.084	0.079	0.037	0.035	0.015
37	Ascending	1.214	0.193	0.191	0.090	0.120	0.051	0.076	0.070	0.033	0.028	0.014
38	Ascending	1.699	0.235	0.217	0.102	0.077	0.036	0.073	0.068	0.031	0.032	0.012
39	Ascending	3.656	0.222	0.202	0.089	0.079	0.054	0.070	0.065	0.032	0.030	0.011
40	Ascending	1.504	0.299	0.297	0.138	0.144	0.055	0.077	0.071	0.035	0.034	0.012
41	Ascending	2.361	0.188	0.183	0.080	0.108	0.031	0.072	0.066	0.032	0.037	0.010
42	Ascending	1.345	0.299	0.291	0.134	0.134	0.049	0.079	0.074	0.036	0.036	0.013
43	Ascending	3.865	0.265	0.259	0.115	0.119	0.065	0.073	0.067	0.031	0.027	0.013
44	Ascending	2.478	0.231	0.230	0.111	0.139	0.033	0.074	0.069	0.031	0.030	0.013
45	Ascending	2.478	0.275	0.268	0.124	0.122	0.043	0.073	0.067	0.029	0.032	0.012
46	Ascending	1.544	0.224	0.206	0.092	0.120	0.058	0.076	0.071	0.031	0.035	0.012
47	Ascending	0.856	0.273	0.271	0.132	0.194	0.064	0.091	0.086	0.039	0.042	0.015
48	Ascending	2.458	0.183	0.182	0.083	0.076	0.029	0.090	0.084	0.041	0.038	0.015
49	Ascending	1.975	0.201	0.196	0.094	0.094	0.044	0.059	0.054	0.026	0.025	0.010
50	Ascending	3.578	0.273	0.260	0.122	0.191	0.051	0.068	0.063	0.028	0.024	0.013
51	Ascending	3.106	0.248	0.246	0.110	0.082	0.057	0.067	0.061	0.028	0.030	0.010
52	Descending	2.033	0.187	0.168	0.073	0.095	0.030	0.072	0.066	0.029	0.031	0.012
53	Descending	3.935	0.231	0.220	0.108	0.102	0.044	0.088	0.083	0.040	0.036	0.016
54	Descending	1.949	0.259	0.242	0.110	0.093	0.050	0.084	0.078	0.035	0.035	0.014
55	Descending	3.346	0.233	0.232	0.106	0.154	0.033	0.065	0.060	0.028	0.030	0.010
56	Descending	2.79	0.271	0.261	0.126	0.193	0.039	0.091	0.086	0.040	0.043	0.014
57	Descending	3.029	0.242	0.231	0.110	0.173	0.039	0.087	0.081	0.037	0.041	0.013
58	Descending	4.812	0.256	0.254	0.112	0.161	0.035	0.082	0.077	0.038	0.036	0.014
59	Descending	2.893	0.301	0.297	0.147	0.145	0.059	0.087	0.081	0.040	0.041	0.014
60	Descending	4.174	0.292	0.273	0.132	0.201	0.057	0.076	0.070	0.030	0.030	0.013
61	Descending	4.389	0.269	0.268	0.130	0.104	0.067	0.063	0.057	0.026	0.029	0.009
62	Descending	1.914	0.235	0.217	0.095	0.161	0.043	0.067	0.062	0.028	0.024	0.012
63	Descending	3.506	0.203	0.202	0.092	0.105	0.049	0.075	0.070	0.034	0.034	0.012
64	Descending	2.021	0.180	0.166	0.070	0.107	0.024	0.080	0.075	0.037	0.039	0.012
65	Descending	2.969	0.282	0.279	0.137	0.156	0.065	0.065	0.059	0.028	0.030	0.010
66	Descending	1.527	0.256	0.247	0.107	0.102	0.061	0.070	0.065	0.031	0.027	0.013
67	Descending	1.874	0.243	0.223	0.110	0.175	0.064	0.085	0.080	0.040	0.045	0.012
68	Descending	3.961	0.261	0.250	0.108	0.176	0.058	0.083	0.078	0.038	0.039	0.013
69	Descending	2.102	0.312	0.301	0.146	0.172	0.067	0.076	0.070	0.031	0.033	0.013
70	Descending	3.186	0.243	0.240	0.100	0.133	0.047	0.082	0.076	0.035	0.040	0.012
71	Descending	1.54	0.209	0.192	0.091	0.152	0.062	0.068	0.062	0.031	0.028	0.011
72	Descending	1.714	0.232	0.215	0.104	0.109	0.038	0.080	0.075	0.036	0.033	0.014
73	Descending	3.023	0.306	0.304	0.145	0.200	0.048	0.086	0.080	0.035	0.038	0.014
74	Descending	1.946	0.222	0.214	0.091	0.116	0.055	0.080	0.074	0.036	0.034	0.013
75	Descending	3.427	0.202	0.196	0.089	0.101	0.032	0.070	0.065	0.030	0.031	0.011
76	Descending	2.136	0.209	0.193	0.083	0.136	0.037	0.074	0.068	0.033	0.032	0.012
**Median**	__	**2.043**	**0.235**	**0.230**	**0.106**	**0.126**	**0.048**	**0.073**	**0.068**	**0.032**	**0.032**	**0.012**

**FIGURE 11:**
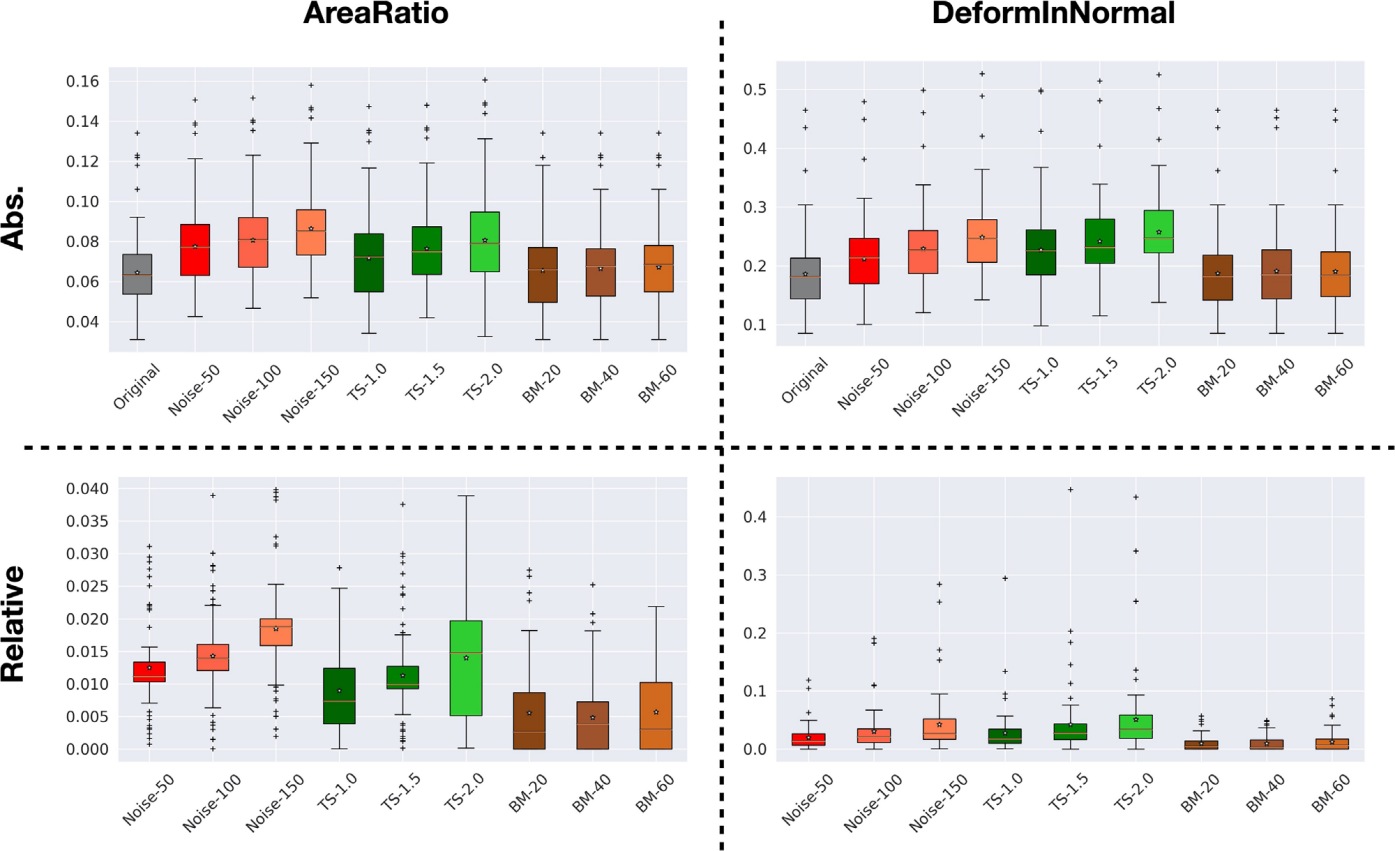
Absolute and relative errors in VDM metrics of aortic growth for all 76 cases. “Original” indicates the VDM result without any perturbations. The reminder of the tests reflect the effects of one graded perturbations in slice noise, thickness of slice (TS), and bulk motion (BM) applied to the fixed and moving images on the VDM outputs. The 99th percentile errors for both AR and DiN is reported, for example, $err=|GT_{\text{AR,DiN}}-VDM_{\text{AR,DiN}}{|}^{99\text{th}}$. The relative error is computed by $\left(err_{\text{perturbed}}-err_{\text{original}}\right)/{GT}^{99\text{th}}$. In the box plots, the “*x*” in the box indicates the mean and line indicates median value. Note that the 99th percentile error is computed without considering the faces and vertices that have deformation smaller than a threshold (0.01 mm)

A summary of the robustness of the AR and DiN measurements to noise, variable slice thicknesses, and bulk motion is shown in Figure 11. In the case of image noise, the 99th percentile error of AR and DiN measurements increased with increasing degrees of image noise; however, the median relative error was 1.4% for AR and 3.3% for DiN at the highest tested noise level (Noise‐150, CNR = 2.66). (Note that the 99th percentile error is computed without considering the faces and vertices which have deformation smaller than a threshold of 0.01 mm). Considering the effects of slice thickness variations, the error similarly increased with thicker slices and was highest at a slice thickness of 2.0 mm, with the highest median relative error of 1.5% for AR and 4.1% for DiN. Bulk motion had a very small effect on measured AR and DiN values with relative errors <1% at all degrees of translation.

**FIGURE 12:**
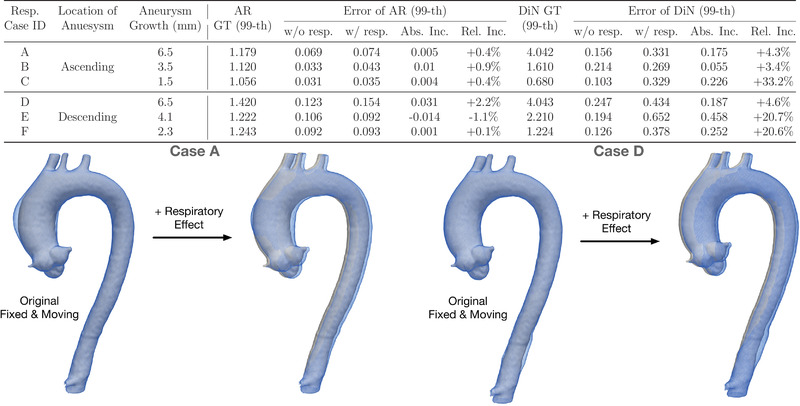
Error in VDM‐based measurements as a function of TAA growth and respiratory translation. The white surface is the fixed surface, while the blue surface is the synthetic moving surface. (99th: 99th percentile. Abs. Inc. = absolute increase. Rel. Inc. = relative increase.)

Results of the six synthetic phantoms combining growth and respiratory motion are shown in Figure 12. Errors were summarized as the 99th percentile error across all vertices on the aortic mesh. The increase in absolute error was computed as the difference in error with and without the presence of respiratory motion. The relative error is computed by dividing the absolute 99th percentile error by the ground truth 99th percentile error. Among synthetic phantoms with the growth of the ascending and descending aorta ranging in magnitude from 1.5 to 6.5 mm the absolute and relative errors associated with respiratory motion were small for AR (maximally 0.031 and 2.2%, respectively). For these same six phantoms, the mean absolute error was 0.23 mm (range: 0.055–0.458 mm).

### 3.2 Comparison between VDM and manual raters

**FIGURE 13:**
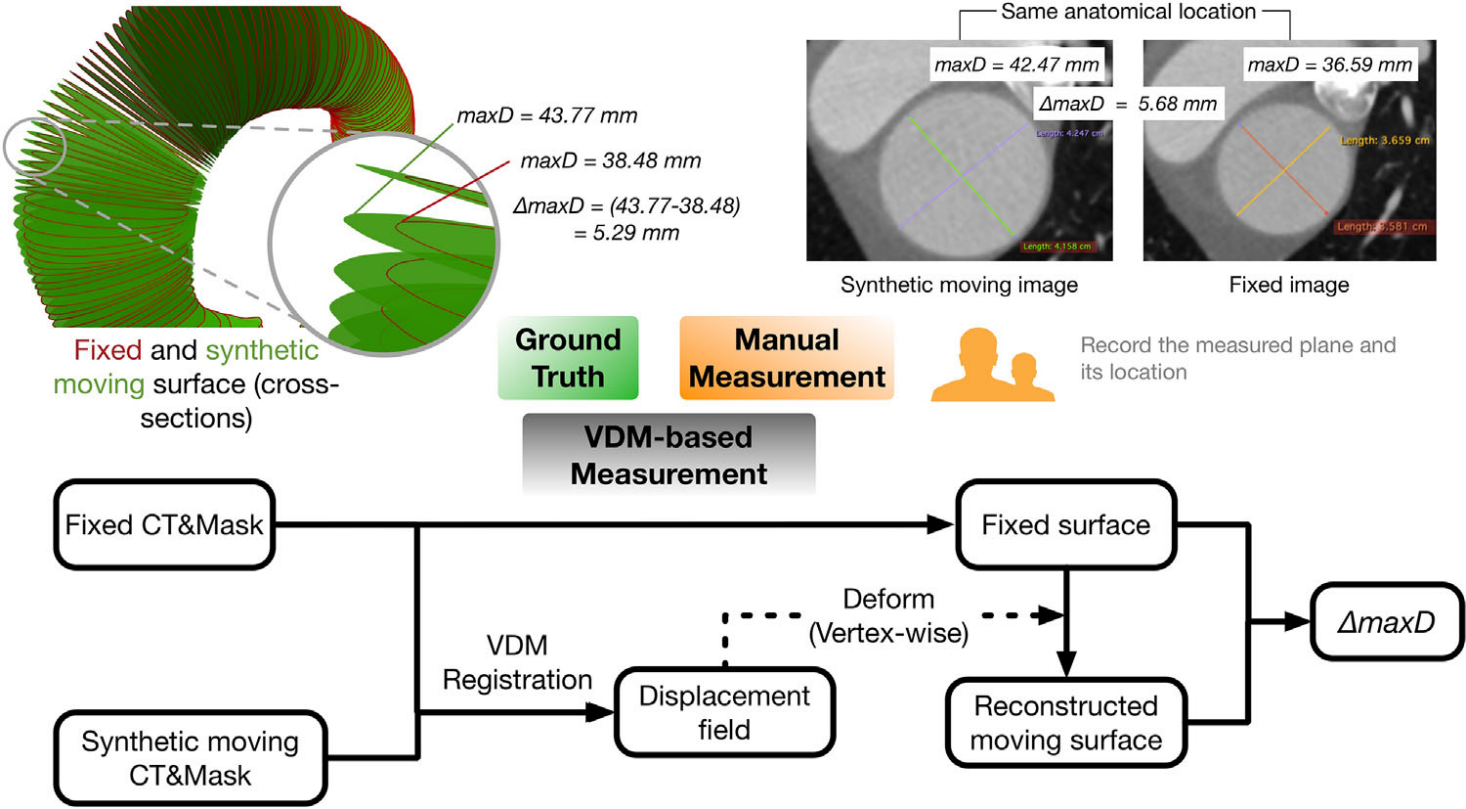
Validation process on maximal diameter change

**FIGURE 14:**
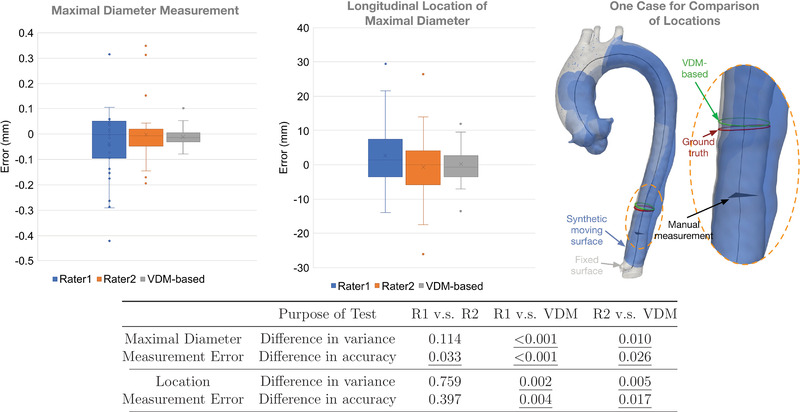
Measurement error of VDM versus manual raters. Two box‐plots on the left show the error in maximal diameter measurements and longitudinal localization by two raters (R1 and R2) and the VDM‐based method. The right figure gives an example of the three locations along the centerline of maximal growth: ground truth location, Rater 1 location (manual), and VDM‐based location. The table below shows the *p*‐values corresponding to comparisons between raters and VDM for testing differences in variance (Levene's test) and accuracy (Wilcoxon test). Statistically significant values (<0.05) are underlined

Following the procedure described in Figure 13, we compared VDM‐based measurements with the manual measurements from two expert raters. Figure 14 shows that the VDM‐based measurements had significantly less variability (i.e., were more precise) than that of the two manual raters and also were significantly more accurate in regard to localization of the area of maximal diameter change. Rater 1 (more experienced) did demonstrate significantly higher accuracy compared to Rater 2 (less experienced) for measurement of the magnitude of maximal diameter change, but there were no significant differences between raters for localization of maximal diameter or variance of diameter measurement error.

## 4 DISCUSSION

Accurate measurement of aortic growth remains an important challenge in the management of patients with TAA. A technique such as VDM that more fully utilizes the 3D nature of aortic CTA data may improve aortic growth assessment by avoiding the variability associated with manually determining the optimal short‐axis plane and making a 1D diameter measurements. In this study, we investigated how the measurement accuracy of VDM compares with manual diameter measurements performed by expert readers and quantified the effects of physiologic and image quality parameters on the measurement performance of VDM. In summary, we found that the DIR‐based VDM‐pipeline was robust to Gaussian image noise and variations in slice thickness (<5% relative error) within the typical range encountered in clinical CTA examinations. Furthermore, we found that VDM‐derived AR measurements were highly robust to physiologic motion of the thoracic aorta due to respiration, although measurement of deformation magnitude in the normal direction demonstrated higher sensitivity to respiratory motion effects. Lastly and perhaps most importantly, we demonstrated that VDM‐derived diameter measurements demonstrated significantly higher accuracy and lower variability in aortic growth measurements compared to manual assessments by expert raters and that VDM was more accurate in identifying the location of maximal aortic growth.

Few prior studies have attempted to quantify aortic growth in a 3D fashion using DIR. Gao et al employed a deformable registration‐based analysis technique, which used a centerline to generate semiautomatic aortic diameter measurements at several discrete locations along the aortic length and compared the reliability of these measurements with manual raters.^16^ However, this study did not attempt to map localized deformation along the surface of the aortic wall and did not employ synthetic phantoms to assess the accuracy of either the semiautomated or manual measurements. As demonstrated in this paper, manual diameter measurements can be significantly variable and inaccurate despite expert raters and an optimal measurement scenario. Specifically, we identified instances where measurement error was up to 3 mm on synthetic phantoms despite excellent image quality, identical CT datasets outside of area of growth, and no differences in patient positioning or physiologic motion. Further, Subramaniam et al. described an approach for quantification of longitudinal aortic growth using contrast‐enhanced magnetic resonance angiography (MRA) in patients with Turner syndrome.^17^ Their technique involved measurement of the Euclidean distance between aortic centerline points and the aortic wall along the length of the aorta, with aortic growth quantified as the differences in these Euclidean distance values between two MRA studies after rigid registration using an iterative closest point algorithm. Similar to Gao et al, Subramaniam et al reported the agreement of their investigational measurements with standard manual diameter measurements, but did not examine the accuracy or robustness of their approach using phantoms, and the accuracy of their approach may be degraded by inaccuracy in segmentation at the aortic boundary and of their point‐cloud based rigid registration. Assessment of measurement accuracy against a reference standard aortic growth/deformation, as performed in this study, is an important step in understanding the real‐world clinical utility of such novel measurement techniques considering the small magnitudes of aortic growth typically encountered in clinical practice (often <2 mm). Similar to previously described techniques, our approach uses aortic segmentation and centerline generation; however, unlike other studies, VDM uses the displacement field (calculated from deformable registration) to deform an aortic mesh. This approach offers several unique advantages including the ability to quantify localized aortic surface area changes and the establishment of point‐to‐point correspondence between baseline and follow‐up aortic geometries. Furthermore, the quantification of aortic wall deformation does not rely on 2D geometric properties such as diameter or Euclidean distance. Despite these advantages, the performance of our new growth metrics (AR and DiN) compared to diameter measurements for predicting clinical patient outcomes remains unclear; however, the AR metric has been demonstrated to have excellent reproducibility in a clinical validation cohort.^4^ Given the multidirectional nature of AR, this metric may better depict mechanical stresses on the aortic wall than 1D diameter measurements.

Using a group of synthetic growth phantoms with realistic shapes, magnitudes, and distributions of growth, we found that VDM measurements of AR and DiN were robust to a variety of image characteristics including image noise and slice thickness with median increases in a relative error being <2% for AR and <5% for DiN at maximal values for Gaussian noise intensity (150) and slice thicknesses (2.0 mm). While medial relative errors were higher with DiN, the absolute magnitude of errors with this metric was still <0.5 mm. We believe the errors encountered in these synthetic experiments are acceptable for routine clinical scenarios given that ECG‐gated CT angiography examinations are commonly reconstructed at slice thicknesses <2 mm and that clinical CT scanners employ dose modulation techniques (e.g., noise index, quality reference mA) to maintain image noise within reasonable limits.^18^ While we acknowledge that Gaussian noise is not a true representation of CT image noise, synthetically generating realistic CT image noise can be a challenging procedure, and we believe that Gaussian noise still allows us to examine the effect on registration accuracy attributable to degrading the signal‐to‐noise ratio at the aortic boundary.

Furthermore, we found minimal error associated with bulk translations/rotations of synthetic CTA pairs (<2% relative error), simulating differences in patient position in the CT scanner between examination, but this is an unsurprising result given that rigid registration techniques are commonly used technique to account for such positional differences. Finally, we found that the errors in AR and DiN values associated with positional changes of the thoracic aorta with respiration (inspiration to expiration), were overall small at physiologic magnitudes,^13^ and while relative errors for DiN attributable to respiratory motion reached 67% maximally, absolute errors were less than 0.46 mm. In clinical practice, we expect these respiratory effects to be even smaller given that our synthetic phantoms simulated the motion associated with peak inspiration to expiration, whereas smaller differences in breath‐hold position would be expected based on standard inspiratory CTA acquisition procedures. Of note, we chose not to systematically evaluate the effects of differing phases of image reconstruction throughout the cardiac cycle (i.e., % R‐R interval), as varying the cardiac phase would instead quantify the effects of pulsatile aortic strain rather than longitudinal aortic wall growth; however, this does assume that the two CTAs used for VDM analysis are reconstructed at the same phase of the cardiac cycle (typically and midlate diastole in clinical practice).^19^

A unique contribution of this paper is the systematic evaluation of measurement accuracy between VDM and manual expert raters of using synthetic phantoms with defined degrees of growth. Multiple prior papers have examined interrater variability of aortic diameter measurements or have compared novel measurement techniques with standard manual measurements; however neither of these approaches, which utilize only clinical data, allow for assessment of measurement error. In an attempt to isolate the effects of measurement error attributable to variability in the location and angulation of measurement planes, we designed our aortic phantom experiment to optimize manual raters ability to produce accurate measurements. Specifically, for these experiments the baseline and follow‐up (deformed) CTAs were identical outside of the area of synthetic deformation eliminating any possibility for differences in contrast timing or image artifacts. Additionally, manual raters told the region (e.g., ascending, descending, or arch) in which the deformation was created, and no bulk translations or rotations were assigned between baseline and follow‐up CTs in this portion of the analysis. Nonetheless, we found that VDM had a significantly lower error in determining maximal aortic diameter change and the location of maximal growth compared to experience manual raters with 5 and 15 years of aortic measurement experience, respectively. While this highly constrained experiment is not a realistic representation of the routine clinical task of aortic diameter measurements, we believe this experimental design highlights the fundamental limitations in 2D diameter measurements for assessing complex 3D aortic anatomy and emphasizes the advantage of a technique such as VDM that more fully utilizes the volumetric CTA data. The measurement errors with manual raters in our study were lower than the typical degrees of measurement variability reported in the literature (± 2 mm on average),^3^, ^15^ ^20^ which probably reflect the highly controlled nature of our experiment.

This study has several limitations. First, our population of synthetic aortic phantoms was created manually using mesh editing software and thus there may be minor geometric differences in patterns and shapes of growth between these phantoms and the morphologies of TAA seen in patients. However, we made substantial effort to generate synthetic growth in realistic locations, patterns and magnitudes based on prior experience with VDM analysis in a clinical TAA population,^4^ and all synthetic phantoms were reviewed by an experienced cardiovascular imager prior to evaluation to confirm only realistic geometries were used. Second, considering that the CTA data used to generate our phantoms was taken from retrospective clinical data, we did not specifically investigate the effects of acquisition (tube voltage/current and pitch) or the specific effects of iterative reconstruction parameters. Third, rather than calculating a displacement field directly from the edited mesh vertices, we employed a simplified B‐spline deformable image registration between boundary images to generate the displacement field from which reference values for AR and DiN were determined. We believe this approach is valid given that we found very small registration errors at this step, and such small errors would have the equal effects on measurement errors for both VDM and manual measurements. Lastly, we did not aim to compare aortic growth in the root (i.e., sinuses of Valsalva) between VDM and manual raters given that the irregular and noncylindrical geometry of this segment makes centerline‐based measurement of maximal aortic diameter unreliable.

## 5 CONCLUSION

Our results confirm that VDM is an accurate technique for 3D assessment of aortic growth in patients with TAA, and is robust to a variety of factors related to image quality and physiologic motion which are present in clinical CTA examinations. Using a group of realistic TAA growth phantoms, we were able to investigate the error of growth assessments in a fashion that is not possible using clinical data, and overall we observed that absolute errors in VDM‐derived measurements of the magnitude of normal deformation and surface area change were less than 0.6 mm and 16%, respectively, across all phantoms and image perturbations. Furthermore, we found that VDM significantly outperformed experienced manual raters in head‐to‐head measurements of the magnitude and location of aortic growth, suggesting that this technique could significantly improve the accuracy and reliability of aortic measurements compared to standard‐of‐care measurement techniques. Further work will be needed to validate the VDM technique in a clinical setting, but these synthetic experiments support both validity of this technique in a controlled setting and provide guidance as to the image and physiologic characteristics that can be tolerated in clinical practice.

## CONFLICTS OF INTEREST

N.S.B. royalties related to the intellectual property of VDM technology studied in this article from Imbio; coinventor of VDM technique (U.S. patent 10,896,507 [techniques of deformation analysis for quantification of vascular enlargement]). Z.B. disclosed no relevant relationships. J.D. disclosed no relevant relationships. J.Z. disclosed no relevant relationships. G.E.C. licensing fees from VDI Diagnostics. C.R.H. Imbio employee, stock in Imbio.

## Data Availability

The data that support the findings of this study are available on request from the corresponding author. The data are not publicly available due to privacy or ethical restrictions.
